# Genome-Wide Association Analysis for Phosphorus Use Efficiency Traits in Mungbean (*Vigna radiata* L. Wilczek) Using Genotyping by Sequencing Approach

**DOI:** 10.3389/fpls.2020.537766

**Published:** 2020-10-29

**Authors:** Venkata Ravi Prakash Reddy, Shouvik Das, Harsh Kumar Dikshit, Gyan Prakash Mishra, Muraleedhar Aski, Surendra Kumar Meena, Akanksha Singh, Renu Pandey, Madan Pal Singh, Kuldeep Tripathi, Padmavati Ganpat Gore, Priti, Twinkle Kumari Bhagat, Shiv Kumar, Ramakrishnan Nair, Tilak Raj Sharma

**Affiliations:** ^1^Division of Genetics, ICAR-Indian Agricultural Research Institute, New Delhi, India; ^2^Division of Plant Physiology, ICAR-Indian Agricultural Research Institute, New Delhi, India; ^3^Division of Basic Science, ICAR-Indian Institute of Pulses Research, Kanpur, India; ^4^Amity Institute of Organic Agriculture, Amity University, Noida, India; ^5^Division of Germplasm Evaluation, ICAR-National Bureau of Plant Genetic Resources, New Delhi, India; ^6^Division of Germplasm Conservation, ICAR-National Bureau of Plant Genetic Resources, New Delhi, India; ^7^Biodiversity and Integrated Gene Management Program, International Center for Agricultural Research in the Dry Areas, Rabat, Morocco; ^8^South and Central Asia, World Vegetable Center, Patancheru, India; ^9^Division of Crop Science, Indian Council of Agricultural Research, New Delhi, India

**Keywords:** mungbean, PUE traits, GBS, GWAS, candidate genes

## Abstract

Mungbean (*Vigna radiata* L. Wilczek) is an annual grain legume crop affected by low availability of phosphorus. Phosphorus deficiency mainly affects the growth and development of plants along with changes in root morphology and increase in root-to-shoot ratio. Deciphering the genetic basis of phosphorus use efficiency (PUE) traits can benefit our understanding of mungbean tolerance to low-phosphorus condition. To address this issue, 144 diverse mungbean genotypes were evaluated for 12 PUE traits under hydroponics with optimum- and low-phosphorus levels. The broad sense heritability of traits ranged from 0.63 to 0.92 and 0.58 to 0.92 under optimum- and low-phosphorus conditions, respectively. This study, reports for the first time such a large number of genome wide Single nucleotide polymorphisms (SNPs) (76,160) in mungbean. Further, genome wide association study was conducted using 55,634 SNPs obtained by genotyping-by-sequencing method. The results indicated that total 136 SNPs shared by both GLM and MLM models were associated with tested PUE traits under different phosphorus regimes. We have identified SNPs with highest *p* value (–log_10_(*p*)) for some traits like, TLA and RDW with *p* value (–log_10_(*p*)) of more than 6.0 at LP/OP and OP condition. We have identified nine SNPs (three for TLA and six for RDW trait) which was found to be present in chromosomes 8, 4, and 7. One SNP present in *Vradi07g06230* gene contains zinc finger CCCH domain. In total, 71 protein coding genes were identified, of which 13 genes were found to be putative candidate genes controlling PUE by regulating nutrient uptake and root architectural development pathways in mungbean. Moreover, we identified three potential candidate genes *VRADI11G08340*, *VRADI01G05520*, and *VRADI04G10750* with missense SNPs in coding sequence region, which results in significant variation in protein structure at tertiary level. The identified SNPs and candidate genes provide the essential information for genetic studies and marker-assisted breeding program for improving low-phosphorus tolerance in mungbean.

## Introduction

The genus *Vigna* includes about 100 species dispersed in subtropical and tropical region globally (Tomooka et al., 2002; Maxted et al., 2004). Among these green gram, black gram (*Vigna mungo*), cowpea (*Vigna unguiculata*), adzuki bean (*Vigna angularis*), moth bean (*Vigna aconitifolia*), creole bean (*Vigna glabrescens*), and tuber cowpea (*Vigna vexillata*) have been domesticated (Takahashi et al., 2016). The wild species are adapted to acid soils, deserts, limestone rocks, sandy beaches, and wetlands (Tomooka et al., 2011). Mungbean is native of India and Central Asia. The closest relative of mungbean is *Vigna radiata* var. *sublobata* (Roxb.) Verdc., occurring in wild state in Western Ghats and Sub-Himalayan tract. From India, mungbean spread to Southeast Asia and through silk road (Tomooka et al., 1992). Mungbean is cultivated in over 7 mha with production of about 5 mt in the world (Nair et al., 2019). The crop is generally grown in East, South, and Southeast Asia. Mungbean is a rich source of protein, minerals, and vitamins (Mubarak, 2005). Mungbean is consumed as *dhal* (whole grain or split) in South Asia and as sprouts, noodles, and paste in East Asia.

Phosphorus (P) is required for photosynthesis, redox reactions, energy production, and phosphorylation/dephosphorylation. The uptake of inorganic P by plants ranges from 10 to 25% (Johnston et al., 2014). Despite being abundant in agricultural soils, the majority of P occurs in an insoluble form (Menzies and Lucia, 2009). P is fixed in acidic soils by metal oxides and by carbonate compounds in alkaline soil or it exists as organic P (Heuer et al., 2017). Low bioavailability of P in soil is due to slow diffusion and high fixation. It is reported that 50% of the world’s arable land is P deficient (Lynch, 2011). Sedimentary deposits are main source of P reserves and account for 80–90% P production. The phosphate rocks are generally located in China, Western Sahara, Morocco, and Russia (Kauwenbergh et al., 2013). These non-renewable, irreplaceable, and finite global phosphate reserves are depleting and are expected to exhaust by the end of this century (Manschadi et al., 2014). The demand for P is increasing to meet the food security of growing population. Environmental problems like water eutrophication and soil degradation result from overuse of phosphorus.

The natural P fertilizer is a limited resource. It is imperative to enhance P fertilizer use efficiency to develop plants with higher uptake and utilization of phosphorous. Phosphorus use efficiency (PUE) measures ability of plant for phosphorus uptake and its use for production of biomass or yield (Wiel et al., 2016). Under P starvation condition, plants activate a range of mechanisms which results in higher uptake of P from soil (Ramaekers et al., 2010). Plant traits increasing P uptake ability includes change in root architecture such as increased lateral root number, root hair length and density, and high root-to-shoot ratio (Lin et al., 2009; Peret et al., 2011). The modification of root architecture along with the exudation of P mobilizing organic acids which results in enhancement of P uptake by plants (Lambers et al., 2006). It is established that vigorous root system with increased nutrient acquisition ability can lead to yield increase under optimized nutrient management system (Vinod and Heuer, 2012). The *Pup1*-specific protein kinase gene and phosphorus-starvation tolerance 1 is reported to be enhancer of early root growth which leads to more acquisition of P in plants (Gamuyao et al., 2012).

The quantitative trait loci (QTLs) for PUE traits in response to P starvation have been reported in crop plants such as wheat (Su et al., 2009; Yuan et al., 2017), rice (Luo et al., 2017; Mahender et al., 2018), corn (Zhu et al., 2005; Chen et al., 2008), common beans (Yan et al., 2004), and soybean (Liang et al., 2010). Conventional linkage mapping utilizes bi-parental mapping populations developed through controlled crosses. Identification of QTLs with small effect requires large population size in bi-parental mapping (Collard et al., 2005). QTL mapping covers small portion of the genetic architecture for a trait as only the allele differing between the parents segregate. Furthermore, bi-parental mapping has less generation and offspring to extensive shuffling of genome and QTLs are localized to a large part of chromosomal regions (10 to 20 cM) (Flint-Garcia et al., 2003).

The association mapping (AM) utilizes panel of diverse genotypes for genome-wide association studies for identification of trait-linked molecular tags. QTLs are identified based on association of marker with trait elucidated by LD between makers and polymorphisms across the AM panel. AM has advantage of the populations undergoing many generations of recombination. Thus, AM provides much finer resolution than genetic mapping. Genome-wide association study (GWAS) have evolved over the last few years into a powerful tool which exploits hundreds of markers across all chromosomes to find genetic variations associated with particular trait (Hirschhorn and Daly, 2005). GWAS and high-throughput next-generation sequencing (NGS) platform make AM as best approach for QTL mapping in plants (Atwell et al., 2010). The genotyping by sequencing (GBS) is a simple and attractive high-throughput multiplexed sequencing method that involves discovery and genotyping of marker (Poland and Rife, 2012). The robustness and low cost of GBS makes this as an outstanding tool for many applications in plants. Since its inception, GBS assay has been successfully applied to develop genetic linkage maps, GWAS, and fine mapping/map-based cloning of QTLs in diverse crops, including soybean (Bastien et al., 2014; Jarquin et al., 2014) and chickpea (Kujur et al., 2015; Upadhyaya et al., 2016).

Single nucleotide polymorphism (SNP) markers are highly desirable for marker-assisted selection (MAS) due to their abundance, locus specificity, codominant inheritance, and high-throughput genotyping (Terakami et al., 2011). Significant SNPs associated with PUE-related traits have been reported in rice (Wissuwa et al., 2015), maize (Wang et al., 2019), cowpea (Ravelombola et al., 2017), oilseed rape (Wang et al., 2017), and soybean (Ning et al., 2016). To our knowledge, to date, a SNP marker associated with PUE traits in mungbean has not been reported. Keeping the importance of P uptake and utilization efficiency traits in mind, the present study aimed to decipher the complex genetic architecture of PUE in mungbean. The study will also lead to advancement of mungbean genomics by enriching the mungbean genome with the development of SNP markers.

## Materials and Methods

### Constitution of Association Mapping Panel and Growing Condition in Hydroponics System

The AM panel for GWAS comprised 144 mungbean genotypes including 40 released varieties, 42 advanced breeding lines (ABL), and 62 exotic germplasm lines (Supplementary Table S1). The experiment was conducted under controlled environment facility at the Indian Agricultural Research Institute, New Delhi, India using hydroponics approach. The seedlings were evaluated in completely randomized design with three replications. In each replication, eight plants per genotype were evaluated. Air temperature in the greenhouse was maintained at 30/18°C (day/night), photoperiod for 12 h, and the relative humidity at 90%. In order to screen genotypes, surfaces of seeds were sterilized with 0.1% (*w*/*v*) HgCl_2_ for 3 min and washed thrice with distilled water to remove detergent. The seeds were kept for germination in germination paper. After emergence of cotyledonary leaves, uniformly germinated seedlings were transferred to modified Hoagland solution with two levels of P. Two P levels were balanced using KH_2_PO_4_ as optimum P (250 μM) and low P (3 μM). The composition comprised CaCl_2_⋅2H_2_O (0.75 mM), K_2_SO_4_ (0.92 mM), Fe-EDTA (0.04 mM), urea (5 mM), MgSO_4_ (1 mM), and micronutrients [H_3_BO_3_ (2.4 μM), ZnSO_4_ (0.6 μM), MnSO_4_ (0.9 μM), CuSO_4_ (0.62 μM), and Na_2_MoO_4_ (0.6 μM)] (Sivasakthi et al., 2017). Nutrient solution was renewed at 2 days interval, and pH was maintained around 6.0 using 1 M HCL or 1 M KOH.

### Phenotypic Evaluation for Phosphorous Use Efficiency Trait in Mungbean

Twenty-one-day-old seedlings grown under optimum- and low-P conditions were carefully separated into leaf, stem, and root to record observations on PUE traits. Isolated complete root system of each plant was placed on a tray with no overlapping of roots for recording observations on root traits. Root traits recorded include total root volume (TRV), total root length (TRL), root average diameter (RAD), total root surface area (TSA), total root tips (TRT), and root forks (RF). These were measured using a root scanner (EPSON XL 1000) with WinRhizo software (Pro version 2016a; Regent Instrument Inc., Quebec, QC, Canada). Primary root length (PRL) was determined manually using meter scale. Chlorophyll concentration (CHL) was measured by using MC-100 chlorophyll concentration meter (Apogee Instruments, Inc., United States). Total leaf area (TLA) was measured using leaf area meter (LI-COR 3000, Lincoln, NE, United States). Separated shoots and roots were dried at 60°C until constant mass of root dry weight (RDW) and shoot dry weight (SDW) was obtained and then root-to-shoot ration (RSR) was calculated. All the traits have been measured in three replications. The PRL and TRL have been measured in centimeters per plant. TSA, TRV, and RAD have been measured in square centimeters per plant, cubic centimeters per plant, and centimeters, respectively. CHL and LA have been measured in micromoles per square meter and square meters per plant, respectively. RDW and SDW have been measured in grams per plant (Table 1). Descriptive statistics, Pearson correlation coefficients, and analysis of variance were performed using the Statistical Tool for Agricultural Research (STAR) 2.1.0 software (Gulles et al., 2014). The broad sense heritability is defined by the formula Vg/Vg + Ve, where Vg and Ve are the genotype and environmental variances, respectively.

**TABLE 1:** Description of the tested traits in the study.

Abbreviated form	Full name	Description	Unit
PRL	Primary root length	The average primary root length of three replications	cm/plant
TRL	Total root length	The average total root length of three replications	cm/plant
TSA	Total root surface area	The average root surface area of three replications	cm^2^/plant
TRV	Total root volume	The average root volume of three replications	cm^3^/plant
RAD	Root average diameter	The average root diameter of three replications	cm
TRT	Total root tips	The average number of root tips of three replications	Number
RF	Root forks	The average number of root forks of three replications	Number
CHL	Chlorophyll concentration	The average chlorophyll concentration of three replications	μmol/m^2^
TLA	Total leaf area	The average leaf area of three replications	m^2^/plant
RDW	Root dry weight	The average root dry weight of three replications	g/plant
SDW	Shoot dry weight	The average shoot dry weight of three replications	g/plant
RSR	Root-to-shoot ratio	The ratio of root dry weight to shoot dry weight	Ratio

### Discovery, Genotyping, Structural, and Functional Annotation of GBS-Based SNPs

The genomic DNA was isolated from the AM panel genotypes. The isolated genotypes were used to constitute two 144-plex GBS library and sequenced (2 × 150-bp single end) using Illumina HiSeq 4000 NGS platform according to Liu et al. (2012) and Bastien et al. (2014). Three accessions were taken as biological replicates. The demultiplexing and mapping of FASTQ reads onto the reference mungbean genome (Kang et al., 2014) had been carried out. The high-quality SNPs were detected using Bowtie v2.1.0 (Langmead and Salzberg, 2012) and reference-based GBS pipeline/genotyping approach of STACKS v1.0^1^ according to Kujur et al. (2015). The unmapped sequence reads were analyzed through *de novo*-based GBS approach of STACKS. Subsequently, the putative SNPs were discovered among genotypes following Kujur et al. (2015). In order to validate SNPs identified through GBS assay, the SNPs present in three candidate genes have been selected. The candidate gene-based SNPs have been validated through Sanger sequencing. Therefore, the primers were designed from 300-bp sequences which were flanking either side of selected SNPs. Next, the PCR amplification carried out using the genomic DNA of 25 mungbean accessions. The 25 mungbean accessions have been selected on the basis of response of root to available phosphorous in soil (Reddy et al., 2020). The purification and sequencing of PCR products were carried out. The alignment of sequences is performed to detect SNPs among accessions according to Kujur et al. (2013).

The reference genome GBS-based SNPs identified in genic and intergenic component of genomes were annotated according to mungbean genome annotation (Kang et al., 2014).

### Construction of Phylogenetic Tree, Determination of Population Structure, and LD Patterns

The genotyping data among mungbean genotypes were analyzed with PowerMarker v3.51 (Liu and Muse, 2005) and MEGA v6.0 (Tamura et al., 2013). An unrooted neighbor-joining (NJ)-based phylogenetic tree (with 1000 bootstrap replicates) had been constructed. The population genetic structure among genotypes was determined by a model-based program STRUCTURE v2.3.4 according to Kujur et al. (2015). The genotyping data of chromosome-based SNPs was analyzed by PLINK and the full-matrix approach of TASSELv5.0 (Saxena et al., 2014). The LD patterns (*r*^2^) and LD decay (by plotting average *r*^2^ against 50 kb uniform physical intervals across chromosomes) had been determined. The LD had been measured as frequency correlation among pair of alleles across a pair of SNP loci.

### Marker Trait Association Analysis

The genome-wide SNP genotyping information of mungbean AM panel was integrated with PUE trait phenotyping data, ancestry coefficient (Q matrix), and relative kinship matrix (K) data using general linear model (GLM) (Q model) and mixed linear model (MLM) approach. The quantile–quantile plot had been generated to determine relative distribution of observed and expected log_10_(*p*) value for each associated SNP. The adjusted *p* value threshold of significance was corrected for multiple comparisons according to false-discovery rate (FDR cut-off ≤ 0.05) (Benjamini and Hochberg, 1995). The SNP loci having significant association with PUE trait at highest *R*^2^ (degree of associated SNP) and lowest FDR adjusted *p* values (threshold *p* < 1 × 10^–5^) were identified by integrating all the model-based outputs of TASSELv5.0.

### Digital Gene Expression Analysis for the Identified Candidate Gene

The digital gene expression analysis has been performed for the candidate genes which have been identified in our study. We have taken the *Arabidopsis* ortholog of candidate gene from mungbean genome for analysis. The gene expression pattern has been searched using Expression Angler, an online search tools (Austin et al., 2016). The experiment had been carried out using different developmental stages like root, shoot, xylem etc.

## Results

### Phenotypic Variation for 12 PUE Traits

Twelve PUE traits were investigated in 144 genotypes of the AM panel under two levels of P conditions. Analysis of variance revealed highly significant variation among 144 genotypes for all tested traits under two P regimes (Supplementary Table S2). We recorded highly significant phenotypic difference between two P regimes (*p* < 0.01) for all traits except PRL, TRV, and TSA, while RDW showed general significance (*p* < 0.05) (Table 2). The mean values of TRL, TSA, TRT, RF, TLA, and SDW were significantly higher under optimum-P condition compared with low-P condition. Whereas, mean values of RAD, CHL, RDW, and RSR were significantly higher in low-P compared with optimum-P condition. The coefficient of variation varied from 3.59 to 15.47% and from 3.32 to 24.31% under optimum- and low-P conditions, respectively. This study showed a maximum heritability (0.92) for TLA under optimum-P condition. At low-P condition, the heritability of CHL in leaf was found to be highest (0.92). The variation in all tested traits under optimum-P condition was significantly correlated with the corresponding traits in low-P condition with Pearson correlation coefficients ranging from 0.39 to 0.76. The trait ratios between low-P and optimum-P conditions ranged from 0.58 (TLA) to 1.78 (RSR) with six values being lower than 1.00 (Table 3). The coefficient of variation for trait ratios ranged from 4.72 to 28.22%.

**TABLE 2:** Descriptive statistical results of phosphorus use efficiency traits of mungbean under different P conditions.

Trait^*a*^	P level	Mean	StDev	Maximum	Minimum	CV (%)	h^2^	*t* value^*b*^	Correlation
PRL	OP	34.88	6.37	49.33	18.67	9.75	0.76	−1.62	0.39***
	LP	36.09	6.36	56.67	22.33	9.39	0.76		
TRL	OP	902.45	206.06	1,714.43	395.97	13.90	0.80	3.91**	0.59***
	LP	814.11	176.31	1,249.23	277.07	13.47	0.69		
TSA	OP	90.08	22.29	188.95	39.82	11.45	0.81	1.66	0.63***
	LP	86.04	19.01	147.06	35.597	13.54	0.70		
TRV	OP	0.726	0.212	1.691	0.348	14.95	0.78	−1.11	0.65***
	LP	0.746	0.187	1.45	0.364	14.92	0.71		
RAD	OP	0.316	0.019	0.370	0.267	4.89	0.63	−9.33**	0.68***
	LP	0.340	0.024	0.427	0.289	4.46	0.73		
TRT	OP	971.84	293.68	2,130.33	418.33	13.87	0.82	5.63**	0.44***
	LP	792.53	244.12	1,480.67	233.67	17.35	0.74		
RF	OP	2,510.79	726.47	4,589.33	998.00	9.18	0.91	8.84**	0.60***
	LP	1,827.26	577.69	3,717.00	448.67	24.31	0.58		
CHL	OP	237.56	25.16	330.33	188.10	3.59	0.89	−19.23**	0.56***
	LP	305.35	34.01	392.67	218.97	3.32	0.92		
TLA	OP	57.01	18.85	136.9	13.85	9.62	0.92	15.70**	0.50***
	LP	30.22	8.01	53.34	9.47	10.40	0.86		
RDW	OP	0.034	0.015	0.110	0.010	15.47	0.90	−1.92*	0.76***
	LP	0.038	0.016	0.107	0.010	16.17	0.89		
SDW	OP	0.187	0.057	0.437	0.080	13.90	0.81	12.54**	0.72***
	LP	0.120	0.028	0.190	0.057	13.36	0.70		
RSR	OP	0.185	0.059	0.384	0.075	13.37	0.85	−12.89**	0.55***
	LP	0.312	0.102	0.569	0.126	14.15	0.83		

StDev, standard deviation; CV, coefficient of variation; h^2^, broad sense heritability; PRL, primary root length; TRL, total root length; TSA, total root surface area; TRV, total root volume; RAD, root average diameter; TRT, total root tips; RF, root forks; CHL, chlorophyll concentration; TLA, total leaf area; RDW, root dry weight; SDW, shoot dry weight; RSR, root-to-shoot ratio; OP, traits tested under optimum-phosphorus conditions; LP, traits tested under low-phosphorus conditions. *p < 0.05; **p < 0.01; ***p < 0.001, levels of significance. ^*a*^The traits tested in the study. ^*b*^Independent *t* test was performed at 1% level of significance to test the significant difference between optimum- and low-P conditions for all tested traits.

**TABLE 3:** Descriptive statistics of trait ratios in 144 diverse mungbean genotypes.

Trait ratio^*a*^	Mean	StDev	Maximum	Minimum	CV (%)
PRL	1.059	0.225	1.750	0.642	15.03
TRL	0.921	0.178	1.427	0.468	18.95
TSA	0.977	0.187	1.574	0.501	18.98
TRV	1.069	0.236	1.808	0.561	22.73
RAD	1.079	0.057	1.214	0.932	6.28
TRT	0.848	0.264	1.789	0.335	22.83
RF	0.746	0.196	1.313	0.321	25.14
CHL	1.291	0.130	1.594	0.992	4.72
TLA	0.568	0.201	1.474	0.247	14.03
RDW	1.163	0.373	2.375	0.583	28.22
SDW	0.668	0.137	1.083	0.366	20.63
RSR	1.780	0.568	3.905	0.757	23.14

*StDev, standard deviation; CV, coefficient of variation; PRL, primary root length; TRL, total root length; TSA, total root surface area; TRV, total root volume; RAD, root average diameter; TRT, total root tips; RF, root forks; CHL, chlorophyll concentration; TLA, total leaf area; RDW, root dry weight; SDW, shoot dry weight; RSR, root-to-shoot ratio. ^*a*^Ratio of tested traits under low to optimum-phosphorus condition.*

### Large-Scale Discovery, High-Throughput Genotyping and Annotation of Genome-Wide GBS- and Candidate Gene-Derived SNPs

**FIGURE 1:**
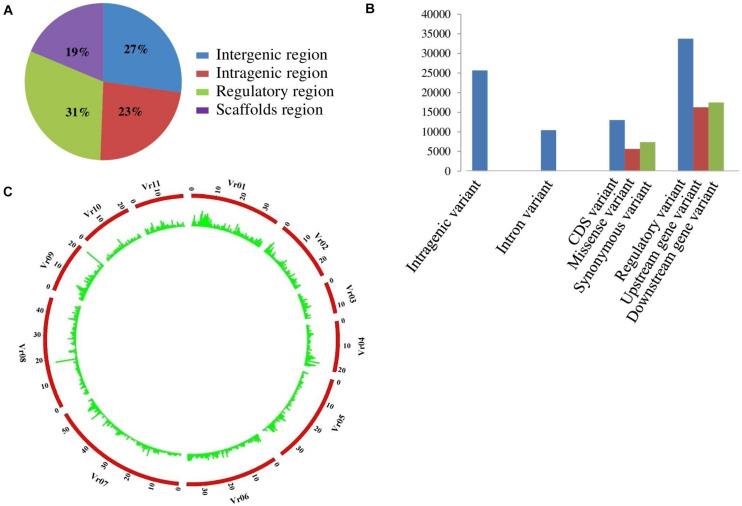
**(A)** Distribution of SNPs in different genomic regions. **(B)** Distribution of large effect SNPs in different genie regions. **(C)** A Circos circular ideogram depicting the genome wide distribution. The outermost circle represents the eleven mungbean chromosomes, while the inner circle represents the relative distribution of SNPs mined from 144 diverse genotypes in mungbean.

A total of 264.40 million high-quality sequence reads had been generated. The reads are evenly distributed (mean, 1.83 million reads) across 144 genotypes of AM panel. Notably, on an average, 75% of these reads were mapped on *V. radiata* reference genome. In total, 76,160 high-quality SNPs (with read-depth 10, <5% missing data, and 8% minor allelic frequency) differentiating 144 samples were discovered using mungbean reference genome-based GBS assay. The genome-wide GBS-based SNP genotyping overall identified 55,634 chromosome-based SNPs showing polymorphism among mungbean genotypes of AM panel. The distribution of all SNPs was found to be in different components of genome including intergenic (27%), intragenic (23%), regulatory (31%), and scaffold (19%) variants (Figure 1A). A total of 20,526 SNPs identified in unanchored scaffold region (Table 4). A maximum of 8,805 SNPs (15.8%) were mapped on chromosome 1, whereas, a minimum of 2,264 SNPs (4.01%) were mapped on chromosome 3. The SNP density was found to be low on chromosome 10 (1.27 SNPs per 100 bp). The structural annotation of 55,634 SNPs identified a number of 25,663 (46.12%) and 29,923 (53.78%) SNPs in the 11,068 protein coding genes (intragenic region) and intergenic regions of mungbean genome, respectively. The abundance of gene-based SNPs was found in the regulatory region (33,724 SNPs, 60.1%), followed by CDS (13,030 SNPs, 23.4%) and least (10,423 SNPs, 18.73%) in the intron region (Figure 1B). In total, 5,643 (43.4% of coding SNP) and 7,387 (56.6% of coding SNP) missense and synonymous SNPs have been found. The relative distribution of GBS-based SNPs mapped on 11 mungbean chromosomes showing polymorphism among AM panel genotypes are presented in a Circos circular ideogram (Figure 1C). The functional annotation of 55,634 SNP-carrying genes revealed that their correspondence lies to 2,481 growth, 7,741 development, 4,936 metabolism-related proteins followed by 764 signal transduction protein. We performed Sanger sequencing-based validation of SNPs present in three candidate genes. Two SNPs present in two candidate genes have been mined through Sanger sequencing (Supplementary Table S4). The structurally and functionally annotated genome-wide SNPs physically mapped on mungbean genome can be used for genotyping applications for quantitative trait dissection of mungbean.

**TABLE 4:** SNP distribution on chromosomes and un anchored scaffold.

Chromosome No.	Size of the chromosome (bp)	No. of SNPs on chromosome	Average density (SNPs per 100 bp)
1	36,501,346	8,805	2.41
2	25,360,630	4,640	1.83
3	12,950,713	2,264	1.75
4	20,812,224	3,724	1.79
5	37,180,910	4,753	1.28
6	37,436,759	6,587	1.76
7	55,601,358	7,290	1.31
8	45,727,239	6,827	1.49
9	21,008,463	4,596	2.19
10	20,996,616	2,671	1.27
11	19,732,206	3,477	1.76
Total	333,308,464	55,634	18.84
Un anchored scaffold	Nil	20,526	

### Molecular Diversity, Linkage Disequilibrium, and Population Structure

**FIGURE 2:**
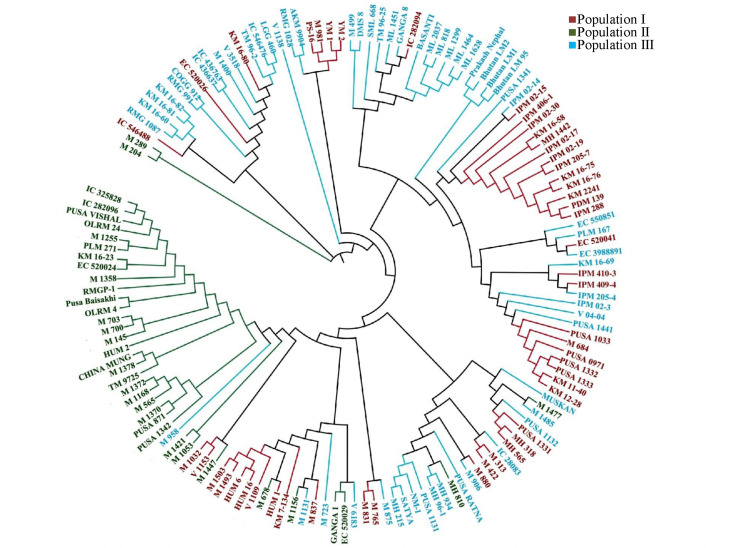
Phylogenetic tree representing the genetic relations among 144 diverse mungbean genotypes based on Nei’s genetic distance using 55,634 high quality GBS based SNPs. Molecular classification differentiated these genotypes into three populations (I, II, and III) based on their origination.

The identified 55,634 genome-wide GBS-based SNPs revealed polymorphism among AM panel mungbean genotypes with higher nucleotide diversity (θπ, 0.35547; θω, 0.18039; and Tajima’s D, 3.21848) potential. Any significant difference for nucleotide diversity value have not been found within the different set of genotype group such as, advanced varieties, landrace, etc. However, we have compared the diversity (θπ) value among the related species, like soybean and chickpea. It has been found that the diversity in soybean was θπ: 0.33 which was comparable to mungbean (θπ, 0.35). In chickpea, θπ was found to be 0.99, which is higher as compared with mungbean. The unrooted neighbor-joining phylogenetic tree construction using 55,634 genome-wide SNPs depicted a distinct differentiation among the AM panel of mungbean (Figure 2). These genotypes were clustered into three major groups.

The LD estimates (*r*^2^) and extent of LD decay were determined using 55,634 SNPs mapped on 11 mungbean chromosomes. We determined the LD decay of 55,634 SNP pairs by pooling the *r*^2^ estimates across 11 chromosomes of mungbean and plotting their average *r*^2^ against the 50-kb equal intervals of physical distance (maximum up to 1,000 kb). The determination of LD patterns in a population of AM panel genotypes using 55,634 SNPs (physically mapped across eleven mungbean chromosomes) showed a higher LD estimate (*r*^2^, 0.62) and less-extensive LD decay (*r*^2^ decreased half of its maximum value) at 50–100 kb physical distance in mungbean chromosomes (Supplementary Figure S3A). A slight increasing and then consistent pattern of LD decay (*r*^2^ ≥ 0.3) was observed with increase in the physical distance (cM) of SNP markers mapped on the 11 chromosomes. The population genetic structure among AM panel genotypes was determined using SNPs (mapped on 11 mungbean chromosomes) employing STRUCTUREv2.3.4 software. The admixture model-based STRUCTURE simulations were performed at different levels of possible population numbers (*K* = 1 to 10) with 20 replications. The population numbers (K) were determined using the second-order statistics of STRUCTUREv2.3.4 by delta K estimation. The results showed a maximum value of delta K at 3 (Supplementary Figure S3B), which confirms the classification of 144 mungbean genotypes into three genetically distinct population groups (POP I–III) with high-resolution population structure (Supplementary Figure S3C). The population I predominantly consists of ABL (23) followed by exotic (17) and released cultivars (9). In population II, exotic (25) genotypes were found to be abundant as compared with ABL (4) and released cultivars (7). In population III, the numbers of released cultivars (24) were found to be higher as compared with exotic (20) and ABL (15). Populations I, II, and III consist of 34, 25, and 41% of total accessions, respectively. In population I, ABL is dominant (47%), population II is dominated by exotic (69%), and population III is found to be dominated by released cultivars (41%). The grouping of all AM panel genotypes into three groups was further confirmed by principal component analysis (PCA) along with phylogenetic tree and population structure analysis (Supplementary Figure S4).

### GWAS for Phosphorous Use Efficiency Traits in Mungbean

For GWAS, the genotyping data of 55,634 GBS-SNPs developed from genotypes of AM panel were integrated with phenotyping data, high-resolution population genetic structure, and PCA data. The Bonferroni-corrected threshold (–log(*p*) = 4.0) was used as a cutoff to identify the significant marker trait associations. A total of 1,171 and 139 significant SNPs were identified by GLM and MLM, respectively. Of these, 136 SNPs were identified by both models (Table 5 and Supplementary Table S3).

**TABLE 5:** Genome-wide association study for 12 phosphorus use efficiency traits studied using two models: the general linear model with Q structure (GLM + Q) and the mixed linear model with Q structure and kinship (MLM).

Condition	Trait^a^	GLM + Q			MLM			*R*^2c^		No. of SNPs shared^d^
		Sig^b^	Average	Range	Sig^b^	Average	Range	Average (%)	Range (%)	
			log_10_(*p*)			log_10_(*p*)				
Optimum P	PRL	64	4.52	4.00–6.75	3	4.40	4.12–4.84	13.74	10.77–19.77	3
	TRL	34	4.89	4.01–7.62	4	4.53	4.23–5.34	14.84	12.27–22.02	4
	TSA	82	4.75	4.01–6.90	7	4.61	4.12–5.16	14.48	12.29–20.18	7
	TRV	94	4.76	4.01–7.18	9	4.60	4.00–5.48	14.52	12.27–20.91	9
	RAD	39	4.49	4.01–5.81	0	–	–	13.64	12.28–17.28	0
	TRT	43	4.46	4.00–6.11	13	4.39	4.04–5.45	13.93	12.25–19.18	13
	RF	84	4.74	4.02–7.51	6	4.32	4.07–5.02	14.36	12.30–21.76	6
	CHL	18	4.36	4.03–5.09	0	–	–	13.28	12.34–15.34	0
	TLA	56	4.67	4.02–8.97	10	4.80	4.02–6.29	14.48	10.37–25.4	10
	RDW	15	5.45	4.05–7.34	10	5.34	4.06–5.99	17.25	12.39–21.32	10
	SDW	61	4.73	4.01–7.50	6	4.91	4.48–5.30	14.54	12.28–21.72	6
	RSR	7	4.80	4.06–6.66	1	5.48	5.48–5.48	14.45	12.42–17.29	1
Low P	PRL	30	4.57	4.03–5.65	1	4.29	4.29–4.29	13.88	12.35–16.86	1
	TRL	35	4.39	4.00–5.39	0	–	–	13.34	12.25–16.16	0
	TSA	45	4.38	4.01–5.52	0	–	–	13.31	12.26–16.49	0
	TRV	18	4.38	4.01–5.62	0	–	–	13.33	12.26–16.77	0
	RAD	8	4.22	4.02–4.43	0	–	–	12.88	12.31–13.47	0
	TRT	11	4.46	4.01–6.29	2	4.53	4.12–4.95	13.42	10.60–20.53	2
	RF	28	4.46	4.02–6.44	7	4.23	4.01–4.93	13.62	12.31–18.98	7
	CHL	18	4.59	4.03–5.72	4	4.44	4.18–4.92	14.16	12.35–17.04	1
	TLA	53	4.42	4.01–5.77	5	4.26	4.03–4.90	13.39	10.33–17.16	5
	RDW	17	4.65	4.17–5.45	10	4.47	4.10–5.11	14.59	12.74–17.84	10
	SDW	50	4.53	4.00–6.82	2	4.42	4.41–4.43	13.54	10.55–19.96	2
	RSR	4	4.46	4.23–4.88	1	4.59	4.59–4.59	13.58	11.22–15.86	1
Ratio	PRL	55	4.49	4.01–5.88	0	–	–	13.45	11.55–17.46	0
	TRL	3	4.36	4.01–4.70	2	4.13	4.07–4.20	13.21	12.09–14.43	2
	TSA	0	–	–	0	–	–	–	–	0
	TRV	0	–	–	0	–	–	–	–	0
	RAD	13	4.36	4.06–5.18	2	4.32	4.17–4.46	13.49	12.42–15.57	2
	TRT	11	4.57	4.00–5.90	6	4.35	4.10–5.24	14.27	12.25–18.42	6
	RF	4	4.26	4.12–4.45	2	4.33	4.25–4.41	12.06	10.76–12.90	2
	CHL	4	4.57	4.52–4.74	1	4.65	4.65–4.65	14.33	13.72–16.18	1
	TLA	72	4.96	4.00–7.90	18	4.43	4.03–5.85	15.02	12.26–22.75	18
	RDW	27	4.63	4.02–6.62	5	4.39	4.24–4.88	14.24	12.29–19.46	5
	SDW	6	4.26	4.08–5.01	1	4.01	4.01–4.01	13.10	12.48–15.11	1
	RSR	62	4.65	4.02–7.28	1	4.43	4.43–4.43	14.09	12.32–21.15	1

*PRL, primary root length; TRL, total root length; TSA, total root surface area; TRV, total root volume; RAD, root average diameter; TRT, total root tips; RF, root forks; CHL, chlorophyll concentration; TLA, total leaf area; RDW, root dry weight; SDW, shoot dry weight; RSR, root-to-shoot ratio. ^*a*^The traits tested in the study. ^*b*^The total number of significant SNPs detected at the threshold of −log(*p*) = 4.0. ^*c*^The phenotypic variation revealed by ANOVA with total significant SNPs detected using the two models. ^*d*^The number of significant SNPs detected by both models.*

**FIGURE 3:**
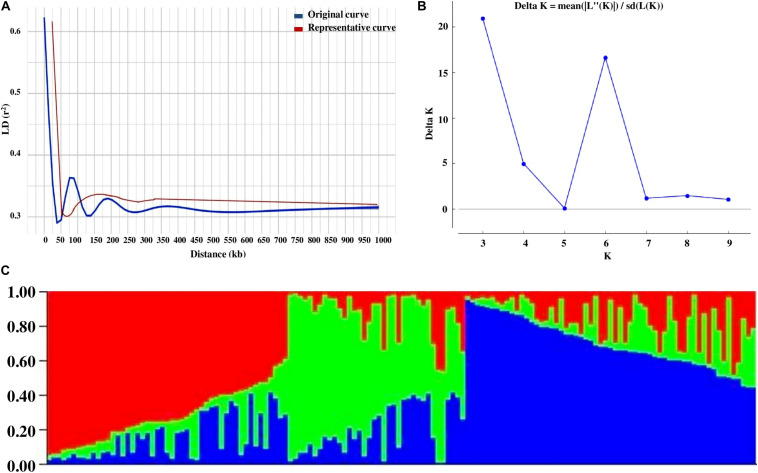
**(A)** Four LD decay measured in association panel of 144 diverse mungbean genotypes. **(B)** Delta K plot showing best peak at *K* = 3. **(C)** Population genetic structure plot of mungbean association mapping panel (optimal population number *K* = 3 with three different colors.

Under optimum-P condition (OP), a total of 597 SNPs identified by GLM were found to be associated with all tested traits in the study. The GLM detected 39 and 18 significant SNPs for RAD and CHL, respectively; for which no significant SNP was detected by MLM. The MLM-detected maximum number of SNP loci (13) was found to be associated with TRT with 13.93% contribution of phenotypic variation. The MLM detected 3, 4, 7, 9, 6, 10, 10, 6, and 1 significant SNPs for PRL, TRL, TSA, TRV, RF, TLA, RDW, SDW, and RSR, respectively (Figure 3 and Supplementary Figure S1). The phenotypic variation (*R*^2^) explained by the SNPs ranged from 13.28 to 17.25%.

**FIGURE 4:**
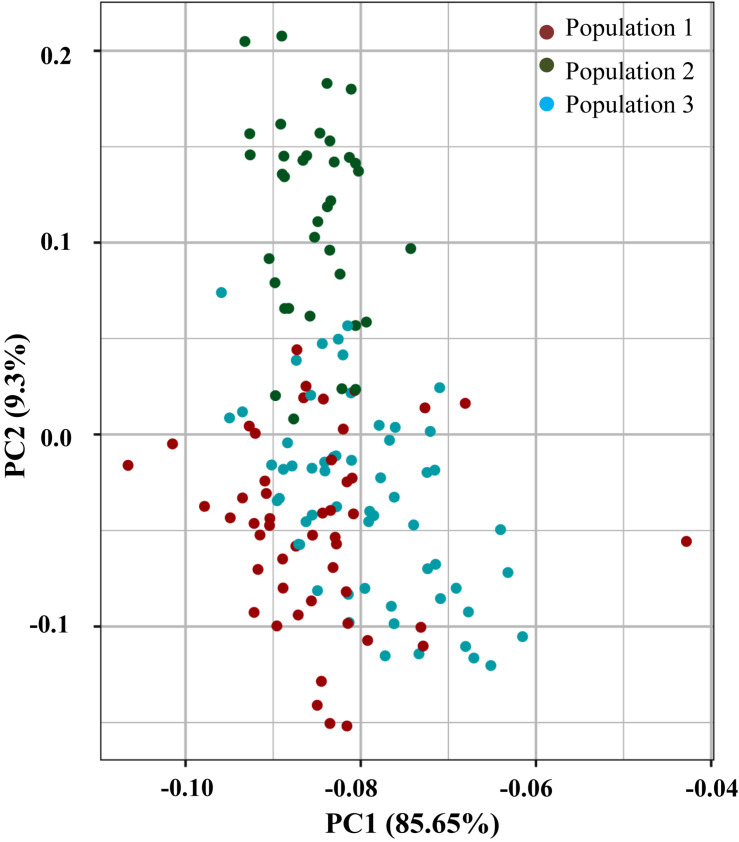
Principal component analysis using 55634 high quality GBS based SNPs assigned to 144 diverse mungbean genotypes into three populations (1, 2, and 3).

Under low-P (LP) condition, a total of 317 significant SNPs associated with tested traits were detected by GLM. GLM detected 35, 45, 18, and 8 SNPs for TRL, TSA, TRV, and RAD, respectively, for which no significant SNP was detected by MLM. MLM detected 1, 2, 7, 4, 5, 10, 2, and 1 significant SNPs for PRL, TRT, RF, CHL, TLA, RDW, SDW, and RSR, respectively. The phenotypic variation explained by SNPs ranged from 12.88 to 14.59%. At LP/OP condition (trait ratios), a total of 257 and 38 significant SNPs associated with tested traits were identified by GLM and MLM, respectively. MLM detected 2, 2, 6, 2, 1, 18, 5, 1, and 1 significant SNPs for TRL, RAD, TRT, RF, CHL, TLA, RDW, SDW, and RSR, respectively. No significant SNPs were identified by both models for trait ratios of TSA and TRV. The phenotypic variation explained by SNPs for trait ratios ranged from 12.06 to 15.02%.

At low-P and optimum-P conditions, MLM detected a maximum number of SNP loci (10) associated with RDW trait with around 14.59 and 17.25% of average phenotypic variation, respectively. Whereas, at LP/OP condition, a number of five SNP loci were found to be associated with RDW trait. At LP/OP conditions, MLM detected a maximum number of SNP loci (18) found to be associated with TLA trait and contributed around 15% of phenotypic variation. Whereas, at OP and LP conditions, a number of 10 and 5 SNP loci were found to be associated with TLA traits, respectively. The association results were further confirmed by analyzing quantile–quantile plot (Supplementary Figure S2). In total, 136 SNP loci were found to be present in a number of 71 protein coding genes. These genes were found to have diverse functions, including in stress, metal ion transport, metabolism, and development. Interestingly, most of the genes identified were found to be involved in nutrient transporting activity. These results suggest the utility of implicating GWAS in mungbean to decipher potential candidate genes for PUE traits in mungbean.

### Delineation of Putative Candidate Genes for PUE Traits in Mungbean

**FIGURE 5:**
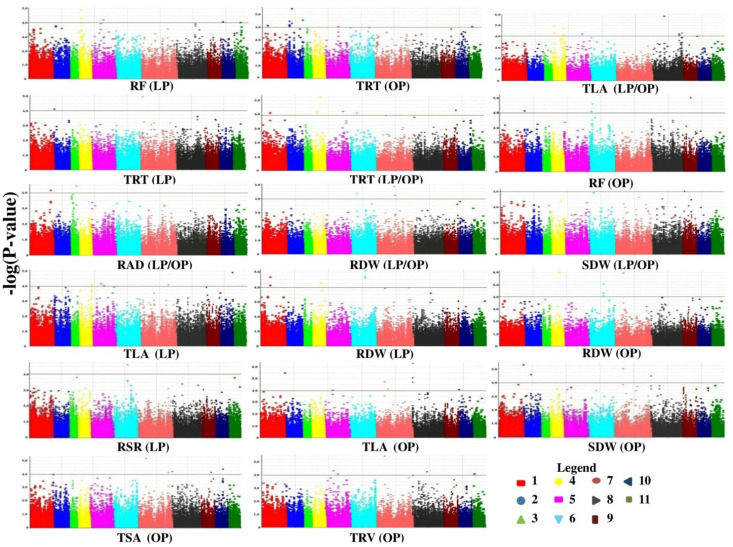
Manhattan plots showing significant *p*-values (measured with mixed linear model) for Phosphorus use efficiency traits associated with 13 putative candidate genes. Colors indicate different chromosomes while horizontal lines indicate a common significance level of *p* = 0.0001 [–log(p) = 4.0]. RF, root forks; TRT, total root tips; TLA, total leaf area; RAD, root average diameter; RDW, root dry weight; SDW, shoot dry weight; RSR, root to shoot ratio; TSA, total root surface area; TRV, total root volume; LP, low phosphorus condition; OP, optimum phosphorus condition; LP/OP, ratio of low phosphorus to optimum phosphorus condition.

In order to delineate the putative candidate genes regulating PUE trait in mungbean, we retrieved the CDS sequences of 71 associated protein coding genes and performed BLAST^2^ search against *Arabidopsis* genome database (with default parameter *p* value ≤ 1.0). The study identified a total of 13 genes with highest similarity and identity (≥80%) to the *Arabidopsis* genes. These genes were well characterized and found to be involved in different nutrient uptake and root architecture development pathway (Table 6). Therefore, we concluded that these genes may be putative candidate genes regulating PUE traits in mungbean. We reported a total of 1,171 and 139 significant SNPs associated with PUE traits by GLM and MLM models, respectively. Of these, a total of 136 SNP loci shared by both models were found to be associated with 71 protein-coding genes (Figure 5). Most of these genes are found to be involved in diverse role, including nutrient uptake, root architecture development, and abiotic stress in mungbean. The *Arabidopsis* orthologous genes are found to be involved mostly in root development and nutrient uptake. Among these 71 protein coding genes, a total of 13 genes are found to be strongly involved in root development and nutrient uptake (Figure 5). The *Arabidopsis* orthologs of these genes are plastidal glycolate/glycerate translocator 1 (*PLGG1*) (*At1G32080*), *At5PTASE9* (*At2G01900*), Ca^2+^-sensor protein kinases-NIN-like protein 7 (*CPK-NLP7*) (*At2G37590*), cellulose synthase-like D gene (*CSLD3*) (*At5G16910*), K^+^ channel *Arabidopsis thaliana* (*KAT1*) (*At4G11480*), ubiquitin-conjugating enzyme E2 13 (*UBC13*) (*At3G46460*), fibroblast growth factor receptor substrate 3 (*FRS3*) (*At2G27110*), GTPase root hair defective 3 (*RHD3*) (*At4G39250*), iron-regulated transport er1 (*IRT1*) (*At5G58440*), *PHO80* (*At5G64260*), K^+^ uptake 7 (*KUP7*) (*At5G09400*), speechless (*SPCH*) (*At5G53210*), and KAN protein (*KANADI*) (*At4G25440*). We found that three genes *VRADI11G08340*, *VRADI01G05520*, and *VRADI04G10750* containing missense SNP at CDS region. These three genes may regulate PUE traits by changing at protein structure level. Therefore, we determined the protein structure by I-TASSER^3^ for these three genes in order to decipher the functional complexity at protein structure level. However, we were able to predict the protein structure for two genes (*VRADI11G08340*; *VRADI01G05520*) (Figure 4). The structural analysis of these genes at protein level revealed a significant variation for native and mutated version of the genes. The missense SNPs present in the CDS of *VRADI11G08340*, *VRADI01G05520*, and *VRADI04G10750* genes changes the amino acids from A/G; methionine to valine, T/C; tyrosine to histidine and T/C; and leucine to serine, respectively. Therefore, it may be concluded that these genes are potential candidate genes regulating PUE in mungbean by changing protein structure at tertiary level. Furthermore, we found that 20 potential candidate genes tended to have pleiotropic effects (Supplementary Table S3). For example, candidate gene *VRADI06G09990* was significantly associated with RDW and RSR under low-P condition, with RDW under optimum-P condition. Similarly, candidate gene *VRADI07G06230* was significantly associated with TLA, RDW, SDW, TRV, and TSA under optimum-P condition. These genes may be involved in many metabolic pathways and affect the PUE traits and therefore worthy of future attention.

**TABLE 6:** Putative candidate genes and their annotation.

Candidate gene	Chr	Associated traits^*a*^	Start position	End position	Definition	Function	References
VRADI11G08340	11	RF (LP)	8932848	8942430	4-Coumarate–CoA ligase-like 6	*At1G32080*; gene *PLGG1* involved in abscisic acid-regulated lateral root development in *Arabidopsis*	Dong et al., 2018
VRADI01G05520	1	TRT (OP)	8450498	8453403	Type IV inositol polyphosphate 5-phosphatase 9-like isoform X2	*At2G01900*; inositol phosphatase genes (*At5PTASE9*) expressed in roots and mediating abiotic stresses in *Arabidopsis*	Golani et al., 2013
VRADI04G10750	4	TLA (LP/OP)	19743783	19754557	Metal binding, zinc	*At2G37590*; transcription factor (*CPK-NLP7*) that regulates the architecture of root and shoot systems in response to changes in nitrogen availability	Gaudinier et al., 2018
VRADI02G01540	2	TRT (LP)	1420232	1425432	Cellulose synthase-like protein D3	*At5G16910*; cellulose synthase-like gene (*CSLD3/KJK/RHD7*) required for cell wall integrity during root hair formation in *Arabidopsis*	Galway et al., 2011
VRADI09G09400	9	TRT (LP/OP)	19095271	19098978	Receptor-like serine/threonine-protein kinase	*At4G11480*; *KAT1* gene involved in regulation of potassium channel activity through abscisic acid signaling, transporter activity by a WNK kinase	Jones et al., 2014
VRADI02G05000	2	TRT (OP)	4861368	4866646	Ubiquitin-conjugating enzyme	*At3G46460*; *UBC13*, ubiquitin conjugating enzyme highly responsive to iron deficiency in *Arabidopsis* by root tips development and auxin signaling	Li et al., 2010
VRADI06G03330	6	RF (OP)	3542363	3550335	Protein FAR1-RELATED SEQUENCE 3-like (*FRS3*)	*At2G27110*; exapted transposable elements in response to phosphate limitation and arsenic toxicity in Arabidopsis	Joly-Lopez et al., 2017
VRADI03G08220	3	RAD (LP/OP)	9791433	9791699	MYB transcription factor MYB142	*At4G39250*; *RHD3* (root hair development 3) proteins mediate endoplasmic reticulum fusion and are essential for plant development in *Arabidopsis*	Zhang et al., 2013
VRADI06G06840	6	RDW (LP/OP)	9440776	9447550	Exocyst subunit Exo70 family protein	*At5G58440*; spatial regulation of *IRT1* expression in iron-deficient *Arabidopsis thaliana* roots	Blum et al., 2014
VRADI09G03030	9	SDW (LP/OP)	3375940	3378249	Protein EXORDIUM-like 2	*At5G64260*; (*PHO80*) represses transcription of genes induced by phosphate starvation	Abercrombie et al., 2008
VRADI10G12490	10	TLA (LP)	19948599	19954318	Potassium transporter	*At5G09400*; Transporter *KUP7* plays crucial roles in K(+) uptake and translocation in *Arabidopsis* root.	Han et al., 2016
VRADI06G09990	6	RDW (LP) RDW (OP) RSR (LP)	21823358	21825860	BHLH domain-containing protein	*At5G53210*; (*SPCH*) initiating stomatal cell lineages during embryo development in soybean	Danzer et al., 2015
VRADI07G06230	7	TLA (OP) RDW (OP) SDW (OP) TRV (OP) TSA (OP)	13822948	13824124	Zinc finger CCCH domain-containing protein 48-like	*At4G25440*; (*KANADI*) regulators of xylem or phloem cell differentiation and activity during secondary growth of root hypocotyl in *Arabidopsis*	Zhao et al., 2005

*Chr, chromosome; RF, root forks; TRT, total root tips; TLA, total leaf area; RAD, root average diameter; RDW, root dry weight; SDW, shoot dry weight; RSR, root-to-shoot ratio; TSA, total root surface area; TRV, total root volume; LP, low-phosphorus condition; OP, optimum-phosphorus condition; LP/OP, ratio of low phosphorus to optimum-phosphorus condition. ^*a*^The key candidate genes associated with phosphorus use efficient traits under different phosphorus conditions.*

### Digital Gene Expression Analysis for the Candidate Genes

The digital gene expression analysis showed that *AT1g32080*, *AT2g01900*, *AT2g27110*, *ATg37590*, *AT3g46460*, *AT4g11480*, *AT5g16910*, *AT5g53210*, *AT5g58440*, and *AT5g64260* gene (ortholog of *VRADI11G08340*, *VRADI01G05520*, *VRADI04G10750*, *VRADI09G09400*, *VRADI02G05000*, *VRADI02G01540*, *VRADI06G09990*, *VRADI06G06840*, *VRADI10G12490*, and *VRADI09G03030*) had the highest expression in lateral root cap, pericycle, lateral root cap, whole root, seed root cell and hairs, whole root, root hair, pollen, seed, and shoot apical meristem, respectively (Supplementary Figure S5).

## Discussion

### Phenotypic P Response of Mungbean Genotypes

The present study showed significant greater root biomass accumulation resulting in higher root-to-shoot ratio under low-P condition. Under P-deficient condition, P-efficient genotype maintained higher root-to-shoot ratio than P-inefficient genotype in maize (Mollier and Pellerin, 1999) and common bean (Nielsen et al., 2001). RDW has been indicated as key trait for determining P starvation response in soybean (Li et al., 2005). Higher chlorophyll concentration in leaves causes dark green foliage under low-P condition. P deficiency causes stunted growth and foliage turns dark green color because of accumulation of starch and sugars in the leaves (Pandey, 2015).

### GBS Assay Accelerate Genomic Resources Development in Mungbean

In the present study, we employed GBS assay for discovery and genotyping of SNPs in mungbean. The length of pseudomolecule assemblies of mungbean genomes was 333.4 Mb representing 57% of total genome. The SNPs mined with GBS assay were found to be dispersed in different portions of mungbean genomes, which may lead to differences in physical map density of SNPs within the genomes. This large number of SNPs (76,160) reported in our study can be utilized for genotyping and QTL mapping for several traits as well as comparative genome mapping involving mungbean and legumes. Additionally, 55,634 chromosome-based SNPs can be used for marker-based applications of mungbean breeding.

### Functional Utility of GBS-Based SNPs in Mungbean

The discovered SNPs were annotated in different components of protein-coding genes of mungbean genomes. The structural and functional annotation revealed that a total of 29,923 SNPs present in intergenic region and 25,663 SNPs in intragenic region. Among the intragenic variants, 10,423 and 13,030 SNPs were found to be present in intron and CDS regions, respectively. Further, the coding variants represented 5,643 and 7,387 missense and synonymous variants. A large number of SNPs were found to be present in regulatory region. The upstream and downstream regulatory region consisted of likely equal number of SNPs (16,260 upstream SNPs, 17,464 downstream SNPs). The structural and functional annotation of genes will accelerate the pace of AM to identify a number of genes/QTLs governing qualitative and quantitative agronomic traits in mungbean. Tajima’s D is a statistical test which is performed to test the null hypothesis of mutation-drift equilibrium and constant population size. A significantly higher Tajima’s D (3.21848) value were observed in our used AM panel. The results indicate that mungbean may undergo for a population reduction (Maruyama and Fuerst, 1985), population subdivision, or a recent bottleneck.

In our study, high-resolution LD pattern were determined in a structured population of 144 mungbean accessions. In soybean and chickpea, several studies have been carried out using GBS and WGRS (Whole genome re-sequence) approach to find the LD pattern and dissect complex trait (Bajaj et al., 2015; Das et al., 2015; Sonah et al., 2015; Zhou et al., 2015; Varshney et al., 2019). In a study, the LD extent was found to be ∼27 kb in wild soybean. In the soybean landraces and improved cultivars, LD was found to be 83 and 133 kb, respectively, which are comparable with mungbean (Zhou et al., 2015). The LD pattern in mungbean is found to be different from chickpea with larger decay at physical position (150–200 kb) (Saxena et al., 2014; Kujur et al., 2015; Upadhyaya et al., 2016; Varshney et al., 2019).

The study revealed a higher LD estimate (*r*^2^, 0.62) and less extensive LD decay with approximately 50–100 kb physical distance in mungbean chromosomes. In a previous study, the LD decay was found to be extensive with approximately 60–100 kb physical distance in a panel of cultivated and wild mungbean accessions (Noble et al., 2018). The LD pattern in mungbean was found to be likely the same to other self-pollinated crop species, like soybean (Patil et al., 2016) but different from chickpea (Saxena et al., 2014; Kujur et al., 2015) with larger decay at physical position. The population structure analysis of the composed panel revealed three population groups which was further confirmed by phylogenetic and principal component analysis. Additionally, this is the first high-resolution determination of LD decay in mungbean with such a large number of genome-wide markers (55,634 SNP markers).

### GWAS to Dissect Complex PUE Traits in Mungbean

The PUE is an important trait which determines yield potential in several crops, including mungbean. Crops with enhanced PUE lead to higher growth for the same amount of P taken up at a time (Rengel and Marschner, 2005). However, the trait is complex and quantitative in nature (Heuer et al., 2017). Therefore, it is desirable to dissect complex genetic inheritance patterns of PUE traits in mungbean. Efforts have been made toward molecular dissection of this complex trait. Several QTLs/alleles for root, shoot, and grain yield-related traits underlying low-phosphorus tolerance have been reported in different legume crop plants including common bean (Beebe et al., 2006; Ochoa et al., 2006; Diaz et al., 2017), Soybean (Song et al., 2014; Zhang et al., 2017) and cowpea (Ravelombola et al., 2017). However, AM has not been explored in mungbean genome to identify functionally relevant molecular tags regulating important traits. In a previous study, a total of 22,000 polymorphic genome-wide SNPs were developed and utilized for AM for seed coat color in mungbean (Noble et al., 2018). In this study, for the first time, such a large number of high-quality SNPs has been developed (76,160 SNPs) in mungbean. Furthermore, these genome-wide well-distributed SNPs have been utilized to perform AM for PUE traits in mungbean. We reported a total of 1,171 and 139 significant SNPs associated with PUE traits by GLM and MLM models, respectively. Of these, a total of 136 SNP loci shared by both models were found to be associated with 71 protein coding genes (Figure 5). Most of these genes are found to be involved in diverse roles, including nutrient uptake, root architecture development, and abiotic stress in mungbean. The *Arabidopsis* orthologous genes are found to be involved mostly in root development and nutrient uptake.

In our study, we identified *bHLH*, *MYB*, and F-box domain-containing and cellulose synthase-like genes. The genes having basic helix-loop-helix DNA binding domains are reported to be involved in root development and biomass accumulation. These genes may help to increase P content and root hair formation (Chen et al., 2007; Li et al., 2011; Giehl and von Wiren, 2014). The genes with *MYB* DNA binding domain is reported to act in controlling architecture of root as well as adaptation to P starvation (Devaiah et al., 2009; Dai et al., 2012; Baek et al., 2013). The F-box domain-containing genes are reported to influence response to low-P stress in crop plants (Baldwin et al., 2008; Perez-Torres et al., 2008; Nakamura et al., 2009). The cellulose synthase-like genes are reported to be involved in cell wall remodeling under low-P condition (Xu et al., 2018). Interestingly, most of the genes exhibited root-specific expression which clearly indicates the possible role of these genes in phosphorous nutrient uptake.

**FIGURE 6:**
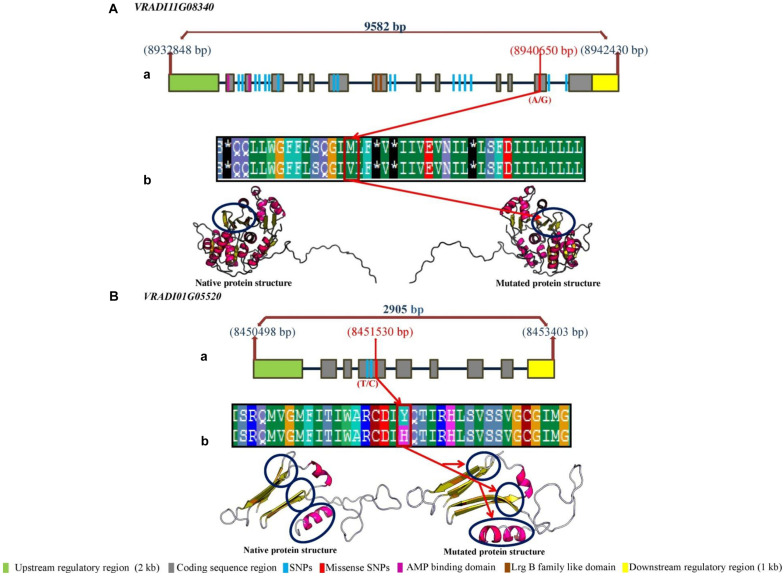
**(A)** (a) Gene structure of potential candidate gene *VRADI11G08340* (b) Conformational changes in protein structure due to functional allele (A/G; missense SNP) at the CDS sequence. **(B)** (a) Gene structure of potential candidate gene *VRADI01G05520* (b) Conformational changes in protein structure due to functional allele (T/C; missense SNP) at the CDS sequence.

Among these 13 putative candidate genes, we identified missense SNPs in CDS region of three genes, *VRADI11G08340*, *VRADI01G05520*, and *VRADI04G10750*. The *Arabidopsis* ortholog genes of these are plastidal glycolate/glycerate translocator 1 (*PLGG1*), inositol polyphosphate 5-phosphatase (*At5PTASE9*), and *CPK-NLP7.* The *PLGG1* encodes a chloroplast protein which regulates lateral root development in response to ABA (Dong et al., 2018). Trull et al. (1997) described the role of ABA in root development under P deficiency with ABA-deficient mutants in *Arabidopsis*. Interestingly, the *AT5PTASE9*, inositol polyphosphate 5-phosphatase is previously reported to be involved in phosphate homeostasis (Kuo et al., 2018; Zhu et al., 2019). The *AT5PTASE9* is a member of the *AT5PTASE* family, which regulates several responses, such as the calcium influx, ROS production, bulk endocytosis, and induction of stress-responsive genes (Golani et al., 2013). The *CPK-NLP7* is found to be involved in a transcriptional regulatory network that regulates the root and shoot architecture in response to changes in nitrogen availability (Gaudinier et al., 2018). The missense SNPs present in the CDS region resulted in structural changes at protein level. Allelic variation of *VRADI11G08340*, *VRADI01G05520*, and *VRADI04G10750* between native and mutated sequences suggests that the respective protein differ in domains and structure for post-transcriptional modification (Figure 6). The differences in protein–protein interactions and signal integration results in differential transcriptional modulations that are causal for observed difference in PUE (Wissuwa et al., 2015). Therefore, these genes may be the potential candidate genes regulating PUE trait in mungbean by changing the protein structure at tertiary level.

## Conclusion

We performed GWAS for PUE traits in a population containing 144 diverse mungbean genotypes with 55,634 SNP markers. The genetic diversity analysis, LD analysis, and population structure of this study will accelerate the pace of mungbean genomics research which can be directed toward genetic improvement of mungbean. The identified SNPs and candidate genes associated with PUE traits can be used in mungbean breeding program to enhance phosphorus deficiency tolerance. These results demonstrated the efficacy and reliability of implementing high-resolution AM in mungbean which has a narrow genetic base and least availability of genomic resources. However, these genes need to be validated further to shed light on their functional role in regulation of the PUE traits in mungbean. Overall, the large number of genomic resources developed in our study will lead to enrich mungbean genomics. These resources can also be utilized to advance genomic-assisted breeding in mungbean.

## Data Availability Statement

The original contributions presented in the study are publicly available. This data can be found here: https://www.ncbi. nlm.nih.gov/sra/PRJNA609409.

## Author Contributions

HD, GM, RP, MS, and VR planned and designed the research. VR, MA, SM, and AS performed the hydroponics experiment. VR, P, TB, KT, and PG helped in data recording. VR, MA, and GM conducted genotyping of the association mapping panel. VR and SD conducted the STRUCTURE, TASSEL, and Bioinformatic analysis. HD, VR, and SD prepared the manuscript. RN, TS, and SK edited the manuscript for publication. All authors have read and approved the final manuscript.

## Conflict of Interest

The authors declare that the research was conducted in the absence of any commercial or financial relationships that could be construed as a potential conflict of interest. The reviewer MKP declared a past co-authorship with one of the authors GM to the handling editor.

## Footnotes

1 http://creskolab.uoregon.edu/stacks

2 https://blast.ncbi.nlm.nih.gov/Blast.cgi

3 https://zhanglab.ccmb.med.umich.edu/I-TASSER

## Supplementary Material

Supplementary Figure 1 Manhattan plots showing significant *p* values (measured with mixed linear model) for phosphorus use efficiency traits.

Supplementary Figure 2 Quantile–quantile plots illustrating the comparison between expected and observed −log_10_(*p*) values to detect significant genomic loci (genes) associated with phosphorus use efficient traits in mungbean.

Supplementary Figure 3 **(A)** LD decay measured in association panel of 144 diverse mungbean genotypes. **(B)** Delta K plot showing best peak at *K* = 3. **(C)** Population genetic structure plot of mungbean association mapping panel (optimal population number *k* = 3 with three different colors.

Supplementary Figure 4 Principal component analysis using 55634 high quality GBS based SNPs assigned to 144 diverse mungbean genotypes into three populations (1, 2 and 3).

Supplementary Figure 5 Digital gene expression analysis and graphical representation of data.

Supplementary Table 1 Source/Origin of 144 genotypes of mungbean used in the study.

Supplementary Table 2 Analysis of variance for the tested traits under two phosphorus regimes.

Supplementary Table 3 List of 136 SNPs associated with PUE traits along with putative candidate genes.
