# Supplementary figures and images for: Genome-Wide Association Analysis for Phosphorus Use Efficiency Traits in Mungbean (Vigna radiata L. Wilczek) Using Genotyping by Sequencing Approach
Source: Front Plant Sci. 2020 Oct 29;11:537766. doi: 10.3389/fpls.2020.537766 (PMC7658405; doi:10.3389/fpls.2020.537766)

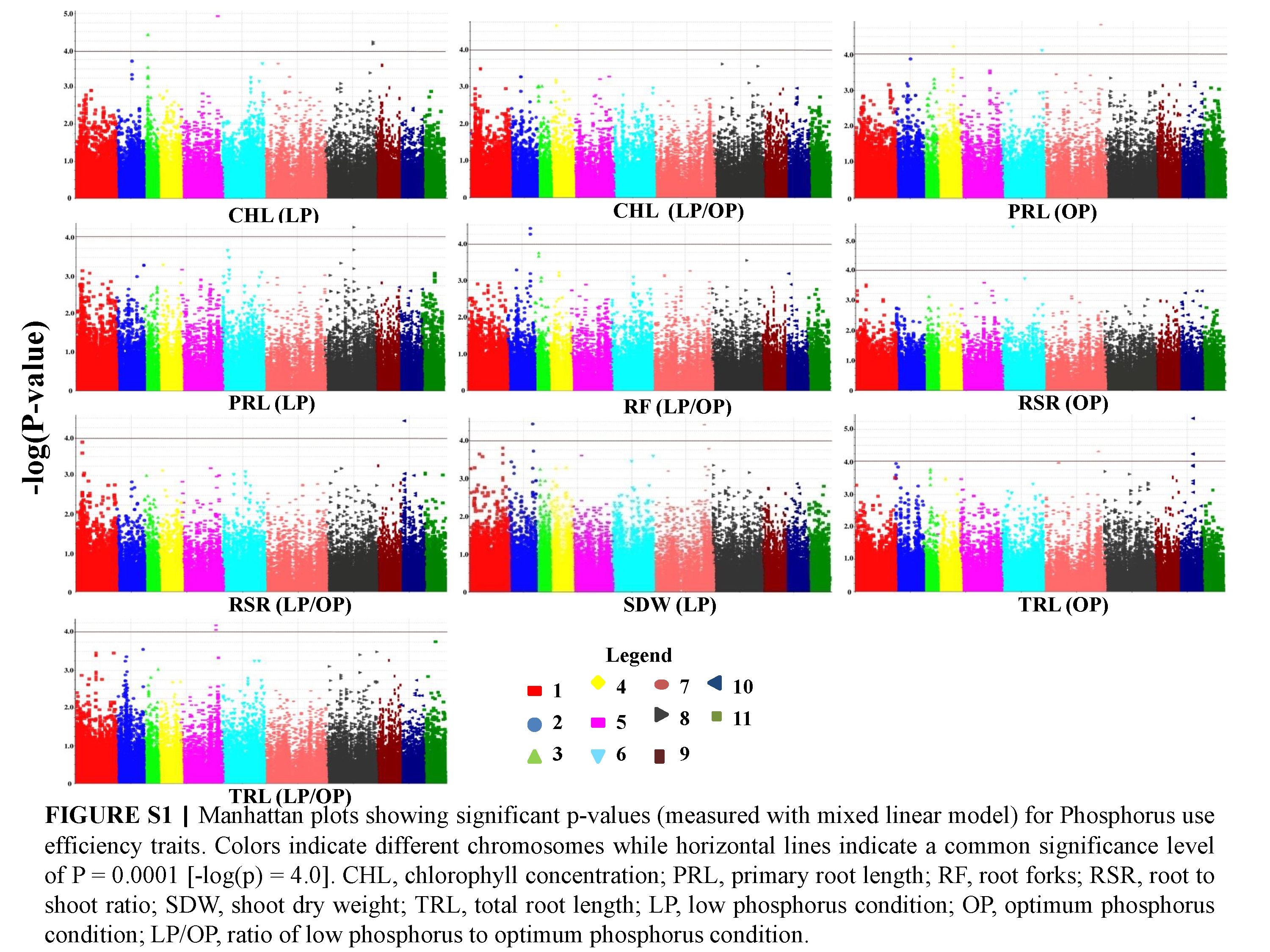

Supplement: Supplementary Figure 1 — Manhattan plots showing significant p values (measured with mixed linear model) for phosphorus use efficiency traits. [file Image_1.JPEG]

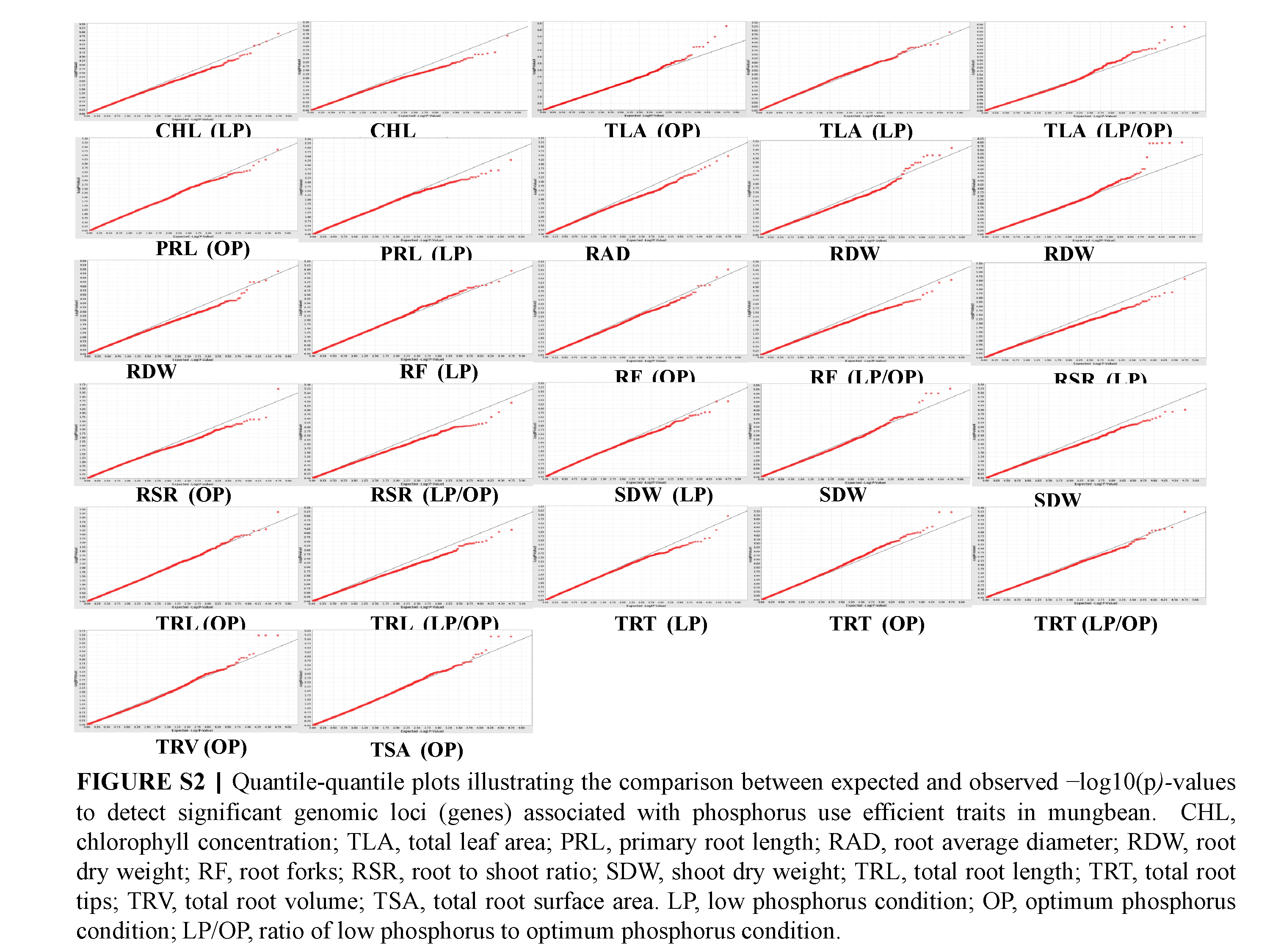

Supplement: Supplementary Figure 2 — Quantile–quantile plots illustrating the comparison between expected and observed −log10(p) values to detect significant genomic loci (genes) associated with phosphorus use efficient traits in mungbean. [file Image_2.JPEG]
