# Supplementary material for: Genome-Wide Association Analysis for Phosphorus Use Efficiency Traits in Mungbean (Vigna radiata L. Wilczek) Using Genotyping by Sequencing Approach
Source: Front Plant Sci. 2020 Oct 29;11:537766. doi: 10.3389/fpls.2020.537766 (PMC7658405; doi:10.3389/fpls.2020.537766)
Supplement: Supplementary Figure 3 — (A) LD decay measured in association panel of 144 diverse mungbean genotypes. (B) Delta K plot showing best peak at K = 3. (C) Population genetic structure plot of mungbean association mapping panel (optimal population number k = 3 with three different colors. [file Presentation_1.PPTX]

## Slide 1
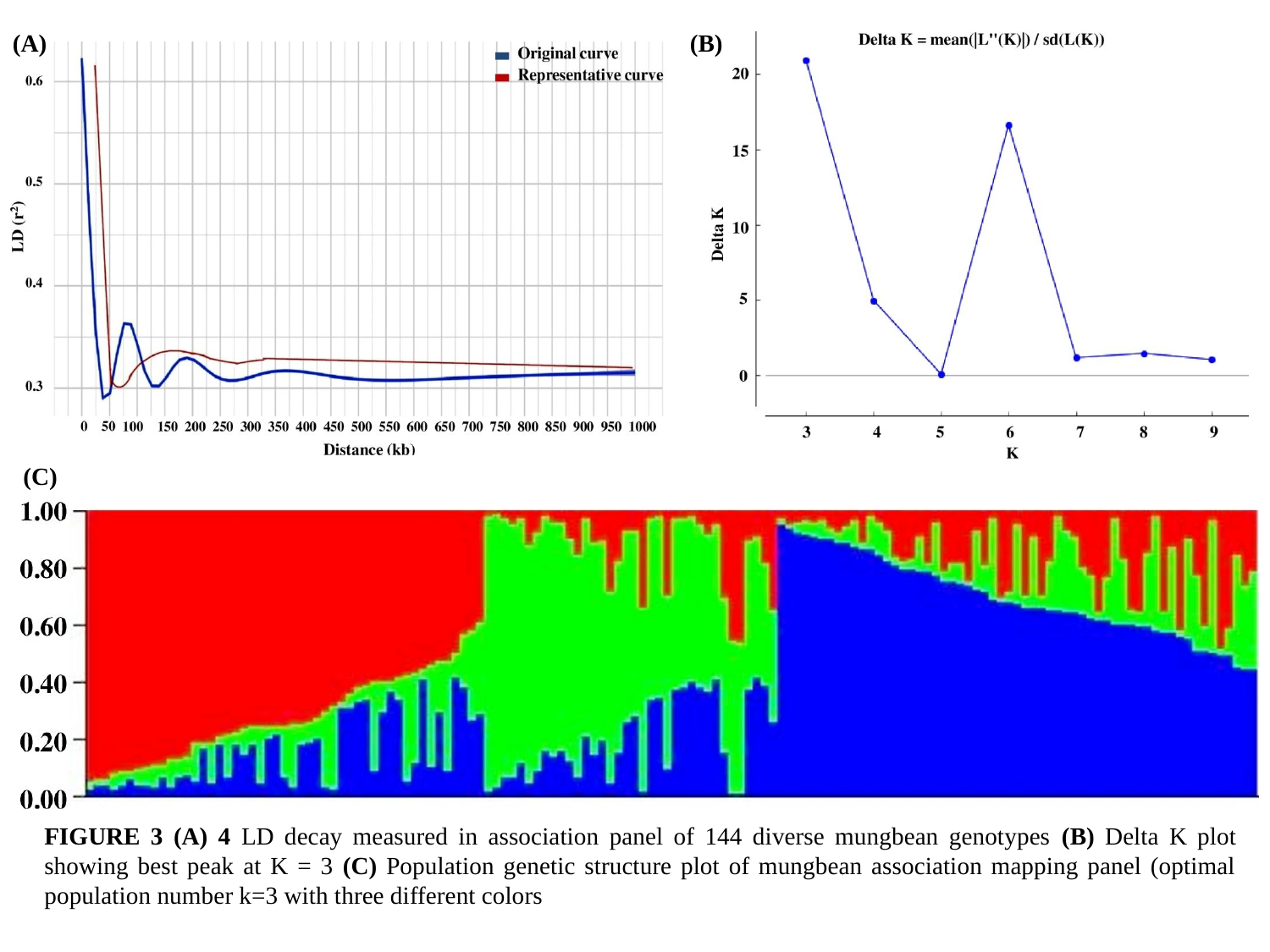

(A)
(B)
(C)
FIGURE 3 (A) 4 LD decay measured in association panel of 144 diverse mungbean genotypes (B) Delta K plot showing best peak at K = 3 (C) Population genetic structure plot of mungbean association mapping panel (optimal population number k=3 with three different colors
