# Supplementary material for: Genome-Wide Association Analysis for Phosphorus Use Efficiency Traits in Mungbean (Vigna radiata L. Wilczek) Using Genotyping by Sequencing Approach
Source: Front Plant Sci. 2020 Oct 29;11:537766. doi: 10.3389/fpls.2020.537766 (PMC7658405; doi:10.3389/fpls.2020.537766)
Supplement: Supplementary Figure 4 — Principal component analysis using 55634 high quality GBS based SNPs assigned to 144 diverse mungbean genotypes into three populations (1, 2 and 3). [file Presentation_2.PPTX]

## Slide 1
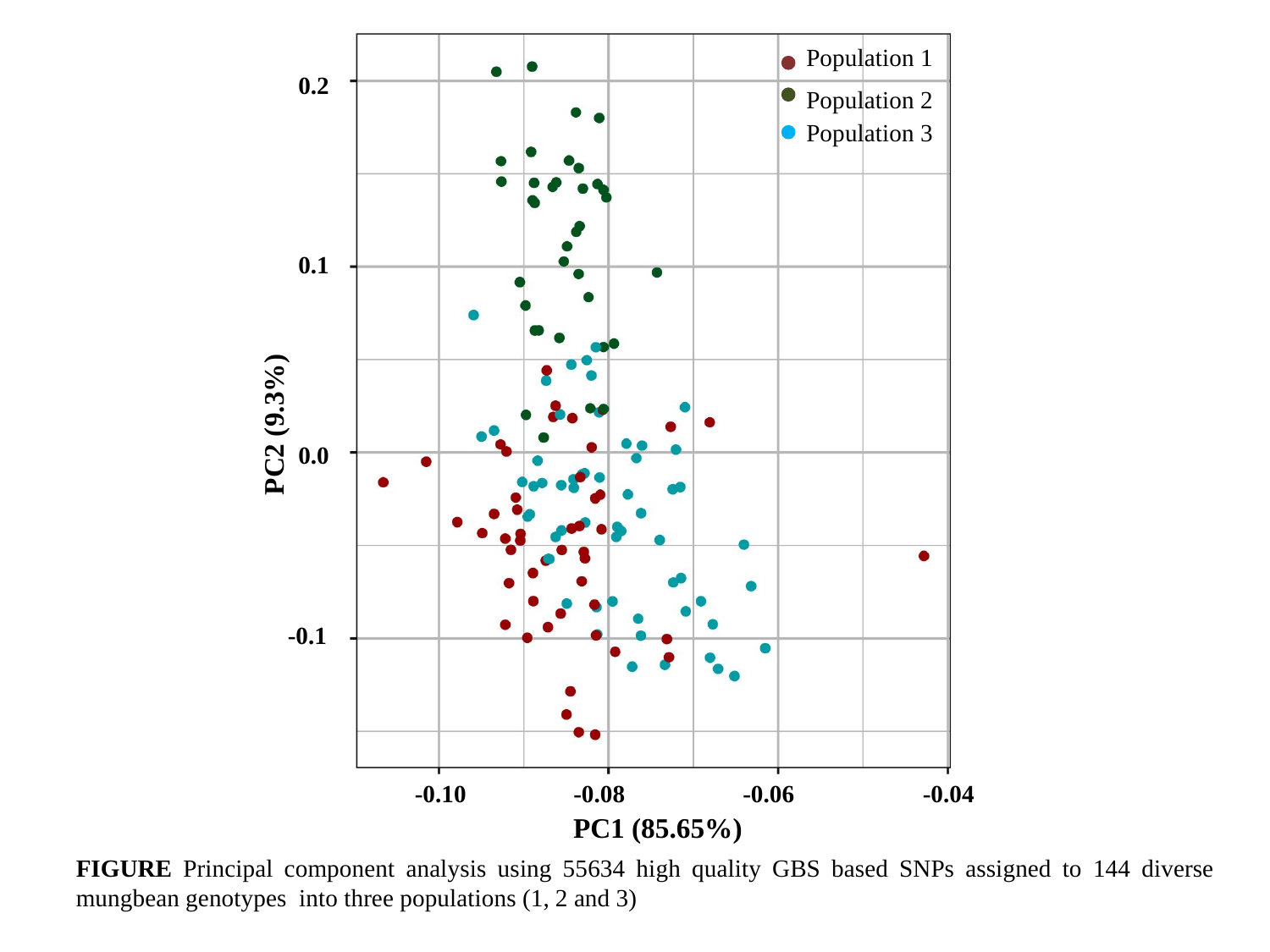

Population 1
Population 2
Population 3
0.2
0.1
0.0
-0.1
PC2 (9.3%)
-0.10
-0.08
-0.06
-0.04
PC1 (85.65%)
FIGURE Principal component analysis using 55634 high quality GBS based SNPs assigned to 144 diverse mungbean genotypes into three populations (1, 2 and 3)
