# Supplementary material for: Genome-Wide Association Analysis for Phosphorus Use Efficiency Traits in Mungbean (Vigna radiata L. Wilczek) Using Genotyping by Sequencing Approach
Source: Front Plant Sci. 2020 Oct 29;11:537766. doi: 10.3389/fpls.2020.537766 (PMC7658405; doi:10.3389/fpls.2020.537766)
Supplement: Supplementary Table 2 — Analysis of variance for the tested traits under two phosphorus regimes. [file Table_2.DOCX]

TABLE S2 **|** Analysis of variance for the tested traits under two phosphorus regimes

| Variables | Mean squares | | | F value | | | Significance | | | CV (%) |
| --- | --- | --- | --- | --- | --- | --- | --- | --- | --- | --- |
|  | G | P | G×P | G | P | G×P | G | P | G×P |  |
| df | 143 | 1 | 143 | 143 | 1 | 143 | 143 | 1 | 143 |  |
| PRL | 168.85 | 318.40 | 74.22 | 14.65 | 27.63 | 6.44 | *** | *** | *** | 9.57 |
| TRL | 174220.65 | 1685666.78 | 46421.16 | 16.06 | 155.39 | 4.28 | *** | *** | *** | 12.14 |
| TSA | 2087.70 | 3541.24 | 488.03 | 17.24 | 29.25 | 4.03 | *** | *** | *** | 12.50 |
| TRV | 0.20 | 0.15 | 0.04 | 16.57 | 12.29 | 3.52 | *** | *** | *** | 14.93 |
| RAD | 0.00 | 0.13 | 0.00 | 10.63 | 553.62 | 2.08 | *** | *** | *** | 4.67 |
| TRT | 313526.47 | 6944862.78 | 123998.72 | 16.92 | 374.76 | 6.69 | *** | *** | *** | 15.43 |
| RF | 2044087.89 | 100917408.17 | 540391.35 | 16.32 | 805.97 | 4.32 | *** | *** | *** | 16.31 |
| CHL | 4114.76 | 992606.26 | 1254.60 | 46.98 | 11331.96 | 14.32 | *** | *** | *** | 3.45 |
| TLA | 856.4125 | 154959.7963 | 401.5863 | 42.90 | 7762.24 | 20.12 | *** | *** | *** | 10.24 |
| RDW | 0.001 | 0.003 | 0.000 | 39.9 | 82.69 | 5.51 | *** | *** | *** | 15.88 |
| SDW | 0.01 | 0.96 | 0.00 | 20.52 | 2063.37 | 5.72 | *** | *** | *** | 14.07 |
| RSR | 0.03 | 3.49 | 0.01 | 24.25 | 2721.02 | 8.52 | *** | *** | *** | 14.40 |

G, genotype; P, phenotype; G×P, genotype × phosphorus interaction; CV, coefficient of variation; PRL, primary root length; TRL, total root length; TSA, total root surface area; TRV, total root volume; RAD, root average diameter; TRT, total root tips; RF, root forks; CHL, chlorophyll concentration; TLA, total leaf area; RDW, root dry weight; SDW, shoot dry weight; RSR, root to shoot ratio.

*, ** and *** significant at P<0.05, P<0.01 and P<0.001 respectively.
